# A refined maximum predictability for next location prediction with fusion knowledge

**DOI:** 10.1371/journal.pone.0342450

**Published:** 2026-02-13

**Authors:** Liuhong Huang, Zhaocheng He, Xiying Li, Zhi Yu

**Affiliations:** 1 School of Intelligent Systems Engineering, Sun Yat-sen University, Shenzhen, Guangdong, China; 2 Guangdong Provincial Key Laboratory of Intelligent Transportation Systems, Shenzhen, Guangdong, China; Sichuan University, CHINA

## Abstract

Research on maximum predictability for next location prediction aims to derive the theoretical maximum accuracy that an ideal prediction model could achieve, which is crucial for analyzing travel regularity and evaluating prediction models. However, three problems remain: 1) The spatiotemporal information used in existing predictability measures is incomplete; 2) quantifying predictability across diverse spatiotemporal information is challenging due to the limitations of entropic measures; and 3) applications of predictability lack further analysis of individual regularity. In this work, we first summarized spatiotemporal information and categorized it into four types of spatiotemporal knowledge. Next, to better quantify predictability, we proposed a refined maximum predictability based on fusion knowledge and Shannon entropy. Finally, we leveraged individual spatiotemporal knowledge preferences based on the refined maximum predictability to analyze travel regularity and evaluate prediction models. Our experimental results showed that the proposed predictability achieved the best results in both the simulation dataset and actual datasets, with a simulation dataset’s mean absolute error (MAE) of 0.06. Furthermore, the evaluation results of prediction models indicated that personalized selection and full utilization of spatiotemporal knowledge are crucial for effective location prediction. This work provides insights into the design and improvement of location prediction models. Codes are available at https://github.com/hlh7/A-refined-maximum-predictability.

## Introduction

Traffic prediction is crucial for decision-making in traffic management [1,2]. As individual next-location prediction is a core component of traffic forecasting, its maximum prediction accuracy dictates the controllable boundaries and potential effectiveness of management strategies. Consequently, understanding this maximum prediction accuracy is essential. The maximum prediction accuracy is also referred to as maximum predictability, representing the theoretical upper bound of prediction accuracy that an ideal prediction model could achieve, independent of particular prediction methods, and reflecting the inherent regularity of individual trips [3]. Maximum predictability is also applied in the analysis of network social activity [4], indoor mobility [5,6], sequential recommendation [7,8], port traffic [9], passenger flow [10], travel time [11], and travel speed [12]. This work focuses on the estimation and application of maximum predictability within next location prediction scenarios.

However, existing methods for estimating maximum predictability in next location prediction, which primarily rely on entropic measures and Fano’s inequality, face several limitations. 1) Incomplete consideration of spatiotemporal information. Some studies focused solely on spatial information, neglecting temporal information [13–16], while others considered only a subset of spatiotemporal information [17–19], leading to an underestimation of predictability; 2) limited by the entropic measures, these methods either struggled to incorporate additional spatiotemporal information [17,18] or incorrectly treated non-repeating trips as regular trips [19], resulting in inaccurate predictability estimations; and 3) the inability to effectively leverage spatiotemporal information prevents accurate predictability estimations across diverse spatiotemporal information, hindering analysis of individual spatiotemporal preferences and insights into travel patterns and prediction models.

To overcome these limitations, this work proposed a refined maximum predictability. First, we examined the compositional relationships within spatiotemporal information and refined the categorization of spatiotemporal knowledge. Second, we enhanced the calculation of the maximum predictability for varying types of knowledge by employing Shannon entropy and random entropy. Third, we integrated predictability across various knowledge to derive the refined maximum predictability and individual spatiotemporal knowledge preferences. Finally, we employed spatiotemporal preferences to optimize group classification and the evaluation and enhancement of prediction models. The experimental results showed that the refined maximum predictability achieved the best results in both the simulation dataset and actual datasets and relatively decreased the mean absolute error (MAE) on the simulation dataset by 68% when compared to the classical method. Moreover, the location prediction model enhanced with spatiotemporal preference achieved comparable accuracy to deep learning models, demonstrating the significant potential of refined maximum predictability. The contributions of this work are as follows.

- We proposed an improved method for estimating maximum predictability. This method fully considers and accurately estimates the predictability across diverse types of mobility knowledge, leading to a more reliable maximum predictability.
- We extended the applications of maximum predictability and applied the refined maximum predictability to mine individual spatiotemporal knowledge preferences, which provides new insights into travel regularity analysis and prediction model evaluations.
- To the best of our knowledge, we were the first to apply individual maximum predictability to improve location prediction models, and the experimental results showed the great potential in the location prediction models based on predictability.

## Related work

### Spatiotemporal information

Spatiotemporal information is the basic input for predictability. In previous studies, the maximum predictability was primarily measured by using the length of the shortest non-repeating subsequence based on location sequences, which utilized destination and origin-destination distributions and ignored temporal information [3,6,13–16]. In addition to spatial information, temporal information is important. Therefore, researchers used the location sequences of time periods to measure predictability by mainly employing time-destination and time-origin-destination distributions [17,18]. In a recent study, predictability was estimated based on the time-origin-destination distribution [19]. However, the spatiotemporal information used in previous studies differs, and no study has provided complete spatiotemporal information.

Studies in other fields can also provide some insights. In addition to predictability, destination [20–23], time-destination [24,25], origin-destination [26], and time-origin-destination [20,26] distributions were used in some studies to determine travel regularity based on entropy. Moreover, the distributions of time [22,27–29], destination [30], time-destination [31], origin-destination [22,32], and time-origin-destination [21,33,34] are usually adopted to mine travel patterns.

Overall, in the study of predictability, four main types of spatiotemporal information related to the destination are present: the distributions of destination, time-destination, origin-destination, and time-origin-destination, which are consistent with those in other related fields.

### Methods of maximum predictability

There are two main tasks in existing studies: the next time-bin prediction task and the next location prediction task. This work focuses on the next location prediction task. Song et al. [3] first adopted real entropy and Fano’s inequality to quantify maximum predictability in the next time-bin prediction task. Wan et al. [13] subsequently extended Song’s method [3] to estimate the maximum predictability in the next location prediction task by applying the Lempel-Ziv algorithm to estimate real entropy based on location sequences. To better estimate maximum predictability, researchers focused on the entropic measures. Some researchers used the Burrows-Wheeler Transform algorithm to estimate real entropy [6,35], which has a higher precision than the Lempel-Ziv algorithm. Xu et al. [36] proposed two suggestions for calculating real entropy through numerical experiments. The real entropy primarily measures maximum predictability based on location sequences, and it neglects temporal information. Thus, the real entropy can quantify predictability well for sequences without any temporal regularity. However, temporal information is crucial in the next location prediction task. For instance, given a sequence similar to postal delivery, like ((t1,d1), (t2,d2), (t1,d1), (t2,d3), (t1,d1), (t2,d4), (t1,d1), (t2,d5)), the location d1 correlates highly with time and is completely predictable; thus, the theoretical maximum predictability can be higher than 0.5. However, according to the real entropy and Fano’s inequality, the estimated maximum predictability is 0.37, which is substantially lower than the theoretical value. Therefore, the real entropy cannot estimate the predictability for temporal information.

To address these problems, Teixeira et al. [17,18] divided the trajectory into multiple location subsequences for time periods and calculated the weighted real entropy based on the subsequences; however, the predictability was mostly underestimated owing to the limitation of the convergence speed. Yu et al. [37] utilized real entropy and mutual information to measure maximum predictability when considering external factors, and they considered exploration trips to be unpredictable; however, those exploration trips have a predictability of 1/*m* when the number of locations is *m*; thus, this method might underestimate the maximum predictability. Consequently, the predictability based on real entropy still cannot quantify the predictability for temporal information well.

To better quantify the predictability for temporal information, Zhang et al. [19] utilized conditional entropy to measure the maximum predictability based on context information. However, this method treats non-repeating trips with a conditional probability of 1 as regular trips, leading to a significant overestimation of predictability. In addition, the conditional entropy usually overestimates the number of locations when utilizing Fano’s inequality, which also results in an overestimation of predictability. These studies indicate that the entropic measures used in existing predictability still have some limitations.

### Applications of predictability

Since Song et al. [3] proposed maximum predictability, many researchers have aggregated individual predictability to analyze group travel regularity, such as average predictability and predictability distributions [3,13–16,19,35,37–43]. Some researchers analyzed predictability distributions for different sampling frequencies and scales and found that predictability correlates highly with sampling frequency and scales in the next time-bin prediction task [38–41]. Wan et al. [13] analyzed the correlations between predictability and the number of visited locations, travel distance, and travel radius, and found negative correlations between them. Ikanovic et al. [14] also investigated the effect of travel features and found that the number of visited locations has the highest correlation with predictability. Yu et al. [37] analyzed the influence of external factors on predictability, such as holidays, temperature, and weather, and found that external factors could increase predictability. Despite the impressive results of group regularity analysis, further individual regularity analysis is needed to enhance the analysis of travel regularity, such as individual knowledge preferences.

In addition, some researchers applied maximum predictability for evaluating prediction models. Some studies analyzed the correlation between predictability and prediction accuracy and found a strong positive correlation between them [19,20,40], indicating that maximum predictability can be used to evaluate prediction models. Furthermore, some researchers evaluated the performance of prediction models for different predictability levels [20,21,44]. However, these evaluations only considered predictability, and other travel features were seldom included, resulting in a single evaluation dimension.

## Problem definition

In this section, we introduce the basic definitions, notations, and problem formulations in this work. Table 1 lists the main notations.

**Table 1:** Summary of notations.

Notation	Description
*u*, *c*, *l*, *t*	user id, category id, location id, time
$s_{n}^{u}$	trajectory with length *n* for user *u*
*L*, *Q*	location set, knowledge set
*D*, *OD*, *TD*, *TOD*	destination, origin-destination, last check-in time-destination, last check-in time-origin-destination
*C^D^*,*C^OD^*,*C^TD^*,*C^TOD^*	D, OD, TD, TOD knowledge
Gd, God, Gtd, Gtod	D, OD, TD, TOD knowledge preference group
$\Pi^{\max}$	maximum predictability
*β*	reduction factor in the threshold of non-repeating trips
$\Pi_{R}^{\max}$	refined maximum predictability

*Definition 1 (Check-in):* Given user *u*, a check-in, also known as a trip, can be defined as $p_{i}^{u}=(l_{i}^{u},t_{i}^{u})$. A check-in contains stay location $l_{i}^{u}$ and check-in time $t_{i}^{u}$.

*Definition 2 (Trajectory):* For user *u*, a trajectory is a time-ordered sequence of check-ins and can be denoted by $s_{n}^{u}=(p_{1}^{u},p_{2}^{u}...p_{n}^{u})$, where *n* is the length of the trajectory.

*Definition 3 (Knowledge set):* The knowledge set can be defined as $Q=\left\{C^{1},C^{2},C^{i},...,C^{k}\right\}$, and each type of knowledge can be denoted by $C^{i}=\left\{c_{1}^{i},c_{2}^{i},c_{j}^{i},...,c_{z}^{i}\right\}$, where *k* is the number of knowledge types, *z* is the number of categories, and $c_{j}^{i}$ is the category *j* of knowledge *i*.

*Problem 1 (Next location prediction):* Given a historical trajectory with length *n* of user *u*, $s_{n}^{u}=(p_{1}^{u},p_{2}^{u}...p_{n}^{u})$, predict the next location, $l_{n+1}^{u}$.

*Problem 2 (Maximum predictability on next location prediction):* Given historical trajectory $s_{n}^{u}=(p_{1}^{u},p_{2}^{u}...p_{n}^{u})$, calculate the maximum accuracy, $\Pi^{\max}$, that the ideal prediction model can achieve in predicting the next location, $l_{n+1}^{u}$.

*Problem 3 (Individual knowledge preference):* For user *u*, given knowledge set $Q=\left\{C^{1},C^{2},C^{i},...,C^{k}\right\}$ and trajectory $s_{n}^{u}=(p_{1}^{u},p_{2}^{u}...p_{n}^{u})$, mine individual travel knowledge that can achieve the highest predictability.

## Proposed method

Because of the incomplete spatiotemporal information and limitations in the entropic measures, the maximum predictability has not been fully considered or accurately quantified for various spatiotemporal information, resulting in a lack of detailed analysis on individual travel regularity. In this work, we first summarized the existing spatiotemporal information to obtain complete spatiotemporal knowledge. Then, we proposed a refined maximum predictability based on Shannon entropy to better quantify the predictability for various spatiotemporal knowledge. Finally, to extend the applications of predictability, we applied the refined maximum predictability to mine knowledge preference, which can be further applied to analyze travel regularity and improve prediction models.

### Spatiotemporal knowledge

Travel time and location are fundamental travel information for predictability calculations. Therefore, this work derives four spatiotemporal knowledge types based on the combined relationships between these two elements. Specifically, in next location prediction, the travel location includes the origin and destination. Thus, there are three key spatiotemporal elements: travel time, origin, and destination. In single-element combinations, the destination-related knowledge contains the destination distribution knowledge. In two-element combinations, destination-related knowledge includes origin-destination distribution knowledge and time-destination distribution knowledge. In three-element combinations, the destination-related knowledge contains the time-origin-destination distribution knowledge. Except for destination distribution, spatiotemporal information has multi-order conditions. For instance, the second-order origin-destination distribution is the destination distribution when considering two previous locations.

In this work, we primarily considered first-order spatiotemporal information. Therefore, the spatiotemporal knowledge set can be defined as $Q=\left\{C^{D},C^{OD},C^{TD},C^{TOD}\right\}$, which denotes the destination, origin-destination, last check-in time-destination, and last check-in time-origin-destination distribution knowledge, respectively. Specifically, we considered two temporal scales: weekly (weekday/workday) and hourly (48-hour periods).

### Refined maximum predictability

Existing maximum predictability is mainly calculated using entropic measures and Fano’s inequality [45,46]. First, an entropic measure is used to estimate entropy based on individual travel information. Then, the entropy is converted into the maximum predictability using Fano’s inequality. Specifically, when the entropy value is *H*, and the number of visited locations is *m*, the maximum predictability, $\Pi^{\max}$, can be obtained by solving the following entropy-predictability-conversion equation:

$$H=-\Pi^{\max}\log_{2}(\Pi^{\max})-(1-\Pi^{\max})\log_{2}(1-\Pi^{\max})\;\;+(1-\Pi^{\max})\log_{2}(m-1)\;$$ (1)

**Fig 1:**
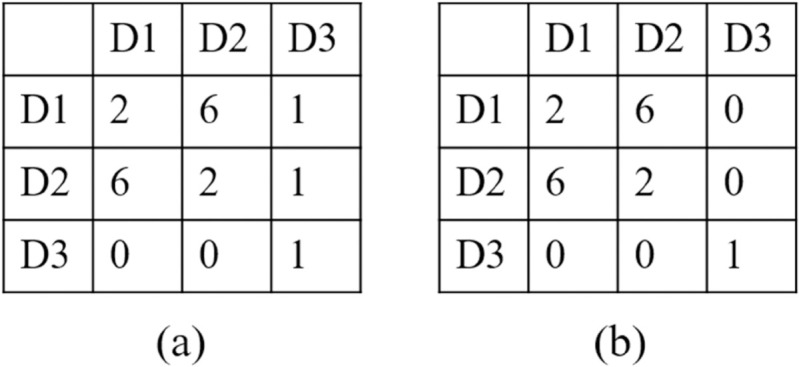
Examples of maximum predictability calculations, and there are three locations, including D1, D2, and D3. (a): Co-occurrence matrix of Individual A. Individual A made single trips for D1 to D3, D2 to D3, and D3 to D3. And Individual A took two trips from D1 to D1 and D2 to D2 and six round trips between D1 and D2. (b): Co-occurrence matrix of Individual B. Individual B’s visits are mostly identical to those of Individual A, with the exception that the frequencies for trips from D1 to D3 and D2 to D3 are both zero.

There are mainly two types of entropic measures: real entropy and conditional entropy. According to Zhang et al. [19], conditional entropy has better expandability than real entropy. However, when non-repeating trips are present, predictability based on conditional entropy is significantly overestimated; for instance, given a location sequence (a, b, c, d, e), the conditional probability of each trip is 1; therefore, the conditional entropy is 0, and the estimated maximum predictability is 1. This indicates that the location sequence is very regular, which is contrary to our knowledge. In addition, conditional entropy usually overestimates the number of visited locations when utilizing Fano’s inequality; for instance, given two individuals’ origin-destination co-occurrence matrices, as shown in Fig 1, there are three locations, including D1, D2, and D3. Individual A takes one trip each from D1 to D3, and D2 to D3. For Individual B, the frequencies for trips from D1 to D3 and D2 to D3 are both zero. According to the conditional entropy and Fano’s inequality, individual A’s maximum predictability is 0.709, while individual B’s is 0.849. Although the differences between the two matrices are negligible, individual B has a much higher predictability than individual A. The key factor is the number of visited locations in Fano’s inequality. In individual B’s matrix, the total number of visited locations is three, but most trips occurred between only two locations. Thus, the number of visited locations is slightly overestimated. Since the maximum predictability increases with the number of visited locations when the entropy value is fixed, the maximum predictability based on conditional entropy is usually overestimated due to the overestimated number of visited locations.

**Fig 2:**
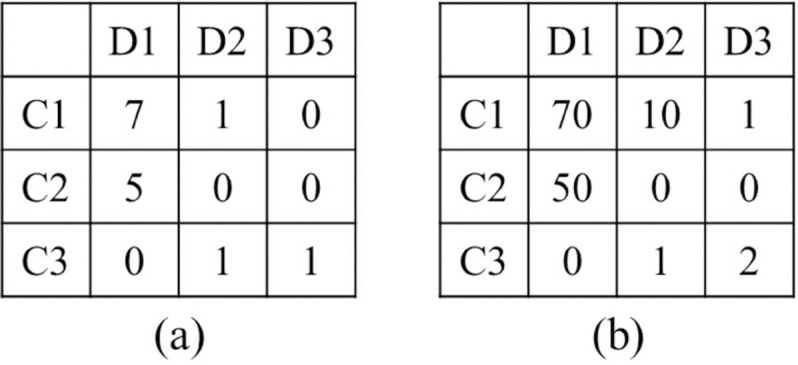
Examples of non-repeating trips. (a): Example 1. Under sub-conditional knowledge C3, the trips to D2 and D3 are both 1, which belong to non-repeating trips, and under sub-conditional knowledge C1 and C2, the trips to D1 are 7 and 5, respectively. (b): Trips to D2 and D3 are 1 and 2 under sub-conditional knowledge C3, respectively. These values are mostly lower than those recorded under sub-conditional knowledge C1 and C2.

To address these problems, we define non-repeating trips as those with low repeatability. For each type of spatiotemporal knowledge, we first categorized the trips into repeated and non-repeating groups. Given a knowledge type, if the maximum destination frequency under sub-conditional knowledge is 1, such as in Fig 2(a) where the maximum destination frequency of sub-conditional knowledge C3 is 1, all trips under this sub-condition can be considered as non-repeating trips. As the number of trips increases, the randomness also increases. For instance, in Fig 2(b), the maximum destination frequency under sub-conditional knowledge C3 is 2, but it is significantly lower than the maximum destination frequencies of other sub-conditional knowledge; trips under C3 still can be considered as non-repeating trips. Therefore, the threshold for classifying non-repeating trips is positively correlated with the number of trips. Additionally, the more visited locations there are, the smaller the threshold should be. Thus, we utilized the individual’s average location frequency and a reduction factor to determine the threshold for non-repeating trips, as shown in Eq 2. The threshold should be greater than zero, and all trips under sub-conditional knowledge with a maximum destination frequency below this threshold are classified as non-repeating trips.

$$Thrf=\max(1,\frac{n}{\beta m})$$ (2)

where *max*() denotes maximum operation, *n* denotes the length of trajectory, *m* denotes the number of visited locations, *β* is the reduction factor and is set to 8 in this work; this is discussed in the next session.

Subsequently, for non-repeating trips, the predictability was set to 1/*m* when the number of visited locations was *m*. For repeated trips, to accurately estimate the number of visited locations, we applied Shannon entropy and Fano’s inequality to measure the predictability for each category set. Finally, the predictability was obtained by weighting the predictability of different category sets according to their category probabilities. Given user *u*, the predictability for knowledge *C^i^* can be defined as:

$$\Pi_{u}^{\max}(C^{i})=\sum_{c\in C_{r}^{i}}P(c)\Pi_{c}^{\max}+\frac{1}{m}\sum_{c\in C_{n}^{i}}P(c)$$ (3)

$$H_{s}^{u}(c{|C}_{r}^{i})=-\sum_{l\in L}P(l|c)\log_{2}(P(l|c))\;=-\Pi_{c}^{\max}\log_{2}(\Pi_{c}^{\max})-(1-\Pi_{c}^{\max})\log_{2}(1-\Pi_{c}^{\max})\;+(1-\Pi_{c}^{\max})\log_{2}(m-1)\;$$ (4)

$$C_{r}^{i}=\{c|\underset{l\in L}{\max}\;(f(c,l))>Thrf,c\in C^{i}\}\;C_{n}^{i}=\{c|\underset{l\in L}{\max}\;(f(c,l))<=Thrf,c\in C^{i}\}\;$$ (5)

where *f*(*c*,*l*) denotes the co-occurrence of location *l* and category *c*, $C_{r}^{i}$ denotes the category set that contains repeated trips, $C_{n}^{i}$ denotes the category set that contains non-repeating trips, *P*(*c*) is the probability of category *c*, P(l|c) is the probability of location *l* when category *c* occurs, *m* denotes the number of visited locations, and *Thrf* is the frequency threshold of non-repeating trips, $\Pi_{c}^{\max}$ denotes the maximum predictability of category *c*, and $H_{s}^{u}(c{|C}_{r}^{i})$ denotes the Shannon entropy of category *c*.

To fully consider all spatiotemporal knowledge, the maximum predictability of different knowledge was selected as refined maximum predictability. Given the spatiotemporal knowledge set $Q=\left\{C^{D},C^{OD},C^{TD},C^{TOD}\right\}$, the refined maximum predictability can be defined as

$$\Pi_{R,u}^{\max}=\underset{C^{i}\subset Q}{\max}\;(\Pi_{u}^{\max}(C^{i}))$$ (6)

We used a case to describe how to calculate the refined maximum predictability. As shown in Fig 1, given individual A’s origin-destination co-occurrence matrix, we considered only the destination distribution and the first-order origin-destination distribution knowledge; therefore, according to Eq 2, the threshold of non-repeating trips is 1. For the origin-destination distribution knowledge, the trips that belong to categories D1 and D2 are repeated trips, while the trips that belong to category D3 are non-repeating trips. The Shannon entropy of the destination distribution knowledge can be calculated as $H_{s}(C^{D})=-16/19\log_{2}(8/19)\;-\;3/19\log_{2}(3/19)=1.47$. According to Eq 1, the predictability, $\Pi^{\max}(C^{D})$, is 0.527. The Shannon entropy of origin-destination distribution knowledge can be calculated as $H_{s}(D1)=H_{s}(D2)=-2/9\log_{2}(2/9)-6/9\log_{2}(6/9)\;-\;1/9\log_{2}(1/9)=1.22$, the predictability is 0.680, and the final predictability of origin-destination distribution knowledge can be calculated as $\Pi^{\max}(C^{OD})=0.68\times 18/19+1/3/19=0.662$. Therefore, the refined maximum predictability is $\Pi_{R}^{\max}=\max(\Pi^{\max}(C^{D}),\Pi^{\max}(C^{OD}))=0.662$.

### Knowledge preference

To extend the applications of predictability, we mined the individual knowledge preference based on the refined maximum predictability. Furthermore, users were then divided into several groups based on the individual knowledge preference, which can be applied to analyze travel regularity and evaluate prediction models.

We mined the individual knowledge preference feature by comparing the predictabilities for different knowledge types and selecting the one with the highest predictability as the individual’s preferred knowledge. Therefore, given the knowledge set $Q=\left\{C^{D},C^{OD},C^{TD},C^{TOD}\right\}$, the predictabilities for different knowledge $S_{\Pi^{\max}}=\{\Pi^{\max}(C^{D}),\Pi^{\max}(C^{OD}),\Pi^{\max}(C^{TD})$, $\Pi^{\max}(C^{TOD})\}$, and the refined maximum predictability $\Pi_{R}^{\max}$, the knowledge preference feature can be defined as $R_{u}=(r_{u}^{D},r_{u}^{OD},r_{u}^{TD},r_{u}^{TOD})$, and each element can be measured using the following equation:

$$r_{u}^{i}=\{1\text{ if }\Pi^{\max}(C^{i})=\Pi_{R}^{\max}\;0\text{ else}\;$$ (7)

There are 15 types of knowledge preference features when considering four types of knowledge, which indicates that users can be divided into 15 groups based on the knowledge preference feature. To better evaluate prediction models, we reduced the number of groups. We set a knowledge priority based on the principle of least knowledge, that is D > OD > TD > TOD. We then categorized the knowledge preference features into four types. Consequently, we obtained four groups: Gd, God, Gtd, and Gtod. Given user *u* and the knowledge preference feature $R_{u}=(r_{u}^{D},r_{u}^{OD},r_{u}^{TD},r_{u}^{TOD})$, the process can be expressed as:

$$Gd=\{u|r_{u}^{D}=1\}\;God=\{u|r_{u}^{OD}=1\text{ and }r_{u}^{D}=0\}\;Gtd=\{u|r_{u}^{TD}=1\text{ and }r_{u}^{D}=0\text{ and }r_{u}^{OD}=0\}\;Gtod=\{u|r_{u}^{TOD}=1\text{ and }r_{u}^{D}=0\text{ and }r_{u}^{OD}=0\text{ and }r_{u}^{TD}=0\}\;$$ (8)

## Experiments

In this section, the datasets and experimental settings are first introduced. Then, we conducted the numerical experiment and case study to evaluate the refined maximum predictability. Finally, the refined maximum predictability is applied to mine individual spatiotemporal knowledge preferences and evaluate knowledge selection and utilization in location prediction models.

### Datasets

We conducted experiments using four real-world datasets: Foursquare NYC (NYC), Foursquare TKY (TKY), Foursquare POA (POA), and Geolife (BJ) [47–50]. The Foursquare datasets were collected from April 2012 to September 2013 in New York, Tokyo, and Porto Alegre using the Foursquare platform. These datasets represent individual real-world POI visit sequences, reflecting diverse visit patterns and featuring a relatively sparse sampling granularity, as they contain only partial individual trips. Conversely, the Geolife dataset was primarily collected from April 2007 to August 2012 in Beijing. It represents a fine sampling granularity, comprising high-frequency GPS trajectories with an average sampling interval of 1-5 seconds. The completeness and accuracy of these datasets are well-established, as they have been widely used in various fields, including travel pattern mining [51–55], next location prediction [56–63], and maximum predictability calculation [19]. For preprocessing, we split the check-ins into multiple sub-trajectories based on an interval of 72 hours, ensuring each sub-trajectory contained no more than 10 records. We then filtered out users with fewer than two sub-trajectories or 10 check-ins. In the location prediction, 80% of each user’s trajectories were used for training, and the rest were used for testing. The preprocessing results are listed in Table 2. The four datasets exhibit significant variability in scale and temporal scope, making them essential for testing method generalizability. The TKY dataset is the largest, featuring the highest number of users, 7341, and records, 741563, over a 10-month period. Conversely, BJ is the smallest in user count, 98, and records, 14566, yet it offers the longest temporal span at 63 months.

**Table 2:** Statistics of the real-world datasets.

Dataset	Users	Locations	Records	Duration
NYC	1080	5133	130952	10 months
TKY	7341	14572	741563	10 months
POA	1838	3778	163347	18 months
BJ	98	172	14566	63 months

Furthermore, each approach has its specific scope. In real-world datasets, imbalanced data and unknown theoretical maximum predictability are common problems. Therefore, a balanced dataset with known theoretical predictability is crucial. To address these problems, we generated a simulation dataset with known theoretical predictability and balanced data. To ensure the simulation dataset reflects real-world mobility behaviors, we primarily followed the approach of Xu et al. [36] and adopted the following strategies. 1) Pattern diversity. The simulation dataset is constructed based on common individual travel patterns: regular and irregular. For regular trips, time-destination and origin-destination patterns are considered, both frequently observed in reality. Irregular trips are modeled as random trips, where individuals randomly select a location from their historical destinations, representing a common form of irregular behavior. 2) Varied regularity. Since individuals exhibit varying degrees of regularity, ranging from predominantly regular to predominantly irregular travelers, we generated a dataset with diverse proportions of regular trips to ensure balanced samples of different regularity individuals. 3) Reduced randomness. To mitigate the impact of randomness, we generated 50 travel sequence samples for each combination of pattern type and regularity level. This reduces the influence of randomness, making the simulation dataset more realistic.

Specifically, we considered two sequence generators with controllable predictability to generate a simulation dataset that contains different pattern types and regularity levels. Given distinct locations *m*, time periods *h*, and length *n*, two types of sequences can be defined as follows.

**Markovian location sequences.** At each step, the location is determined by a fixed location transition order (i.e., a, b, c, d, a, b, c, d... ) with probability *p* (representing regular origin-destination trip), and the location is randomly selected from *m* candidate locations with probability 1–*p* (representing irregular trip); therefore, the theoretical maximum predictability is p+(1−p)/m.

**Markovian time-location sequences.** At each step, the location is determined based on fixed time-location transition rules (i.e., (t1, a), (t2, b), (t1, a), (t3, c), (t3, c), (t2, b)... ) with probability *q* (representing regular time-destination trip), and the location is randomly selected from *m* candidate locations with probability 1–*q* (representing irregular trip); therefore, the theoretical maximum predictability is q+(1−q)/m.

We set the number of locations *m* to 10 and 20, respectively, and set length *n* to 200 and number of time periods *h* to 48 based on the work conducted by Xu et al. [36]. Furthermore, to diversify the regularity degrees in the simulation dataset, we set the interval to 0.1 and adjusted *p*, *q* in the range of [0, 1]. Finally, to reduce randomness, we generated 50 sequences for each *p* and *q* to form a simulation dataset containing 2200 trajectories.

### Experimental settings

**Evaluation metric.** To evaluate the performance of the location prediction, the standard accuracy metric (Top K) [64] was used, which shows the ground-truth in the top K prediction results. The MAE was selected to measure the mean absolute error between the theoretical predictability and estimated predictability.

**Next location prediction models.** We selected Markov, HSTLSTM [64], DeepMove [65], LSTPM [66], and MHSA [67] as location prediction models. Specifically, we used the first-order Markov model, and the other models are mainstream deep learning methods. Furthermore, for each user, we selected the maximum accuracy among the prediction models as the maximum accuracy. The test results on the real-world datasets are presented in Table 3. The LSTPM model achieved the highest prediction accuracy, recording 0.248 on the NYC dataset and 0.266 on the POA dataset. For the TKY dataset, DeepMove delivered the top result at 0.208, while MHSA proved most effective on the BJ dataset, reaching an accuracy of 0.557.

**Table 3:** Performance of location prediction models on the real-world datasets.

Model	NYC			TKY		
	Top1	Top5	Top10	Top1	Top5	Top10
Markov	0.232	0.485	0.576	0.178	0.355	0.417
HSTLSTM	0.195	0.439	0.523	0.150	0.324	0.396
DeepMove	0.243	0.548	0.657	**0.208**	0.428	0.507
LSTPM	**0.248**	**0.559**	**0.667**	0.193	0.421	0.512
MHSA	0.238	0.550	**0.667**	0.203	**0.443**	**0.540**
**Model**	**POA**			**BJ**		
	**Top1**	**Top5**	**Top10**	**Top1**	**Top5**	**Top10**
Markov	0.217	0.419	0.508	0.535	0.857	0.912
HSTLSTM	0.211	0.406	0.473	0.520	0.849	0.894
DeepMove	0.261	0.551	0.636	0.549	0.882	0.917
LSTPM	**0.266**	0.549	0.632	0.556	**0.887**	**0.922**
MHSA	0.261	**0.555**	**0.647**	**0.557**	0.879	0.914

**Comparison of maximum predictability.** We compared our method with five state-of-the-art baselines: 1) real entropy (RE) [13–16], which mainly measures the maximum predictability based on the location sequences; 2) refined real entropy (RRE) [36], which improves the calculation process of real entropy; 3) conditional entropy (CE) [19], which measures the maximum predictability based on the distribution of time (48 h)-origin-destination; 4) fusion conditional entropy (FCE) [68], which utilizes conditional entropy to calculate the entropy for different knowledge and selects the minimum value among them as the minimum entropy. And we applied the fusion conditional entropy and Fano’s inequality to the complete spatiotemporal knowledge; 5) fusion multivariate sample entropy (FMSE): We applied the multivariate sample entropy [69–71] to the complete spatiotemporal knowledge and selected the maximum predictability derived from different knowledge types as the final result.

### Evaluation of maximum predictability

We primarily conducted two experiments to validate the efficacy of our refined maximum predictability. We first evaluated various predictability methods by calculating the mean absolute error (MAE) between their estimated results and the theoretical ground truth in the simulation dataset. Then, using real-world datasets, we assessed their performance by analyzing the correlation between the estimated predictability and the corresponding maximum prediction accuracy.

**Fig 3:**
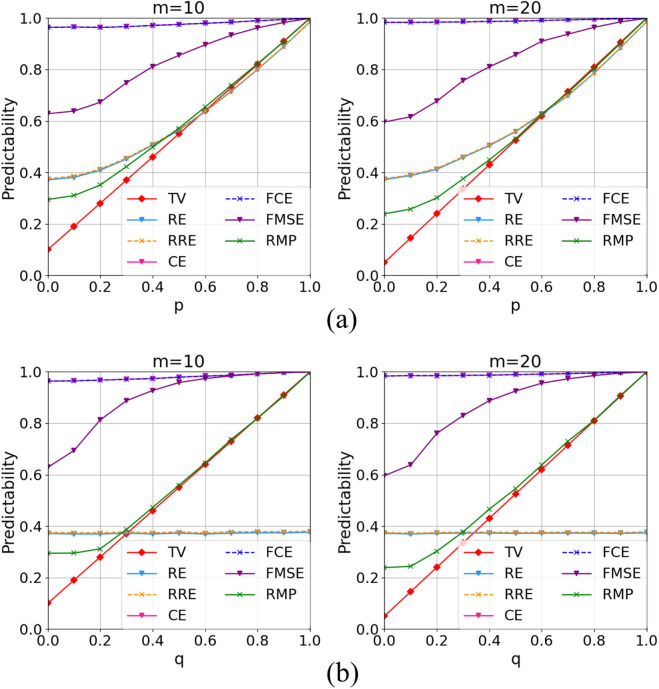
Predictability based on simulation dataset. (a): Predictability based on Markovian location sequences. (b): Predictability based on Markovian time-location sequences.

*1) Numerical experiment.* Since the real-world datasets exhibit sample imbalance and lack ground truth for maximum predictability, we utilized the simulation dataset with theoretical maximum predictability and balanced samples to verify the performance of the refined maximum predictability. Specifically, the performance of predictability calculation methods was evaluated by comparing their results to the theoretical maximum predictability value (TV), with smaller errors indicating better performance. As shown in Table 4, the refined maximum predictability yielded the lowest MAE across all experimental configurations, ranging from 0.058 to 0.066, whereas the real entropy and refined real entropy achieved the lowest MAE among all baselines, which ranged from 0.095 to 0.286. Overall, the refined maximum predictability relatively decreased the total MAE by 68% compared to the state-of-the-art methods. Specifically, as shown in Fig 3, on Markovian location sequences, the refined maximum predictability achieved much lower MAEs than real entropy for users with low predictability. On Markovian time-location sequences, as the real entropy and refined real entropy cannot capture the predictability for temporal knowledge, they maintained the same predictability value for individuals regardless of their temporal regularity. In contrast, our proposed method effectively captured the predictability for individuals with differing levels of temporal regularity. In addition, because of the non-repeating trips, the fusion conditional entropy obtained the highest predictability when considering the time (48 h)-origin-destination distribution knowledge. Thus, the predictabilities based on conditional entropy and fusion conditional entropy were equal and considerably higher than the theoretical value. These findings suggest that the proposed refined maximum predictability provides a significantly more accurate estimation of individual predictability in next location prediction compared to traditional methods.

**Table 4:** Performance of different maximum predictabilities on simulation dataset.

Method	Markovian location sequences		Markovian time-location sequences		Total
**Locations (m)**	**10**	**20**	**10**	**20**	
Real Entropy	0.095	0.118	0.277	0.286	0.194
Refined Real Entropy	0.097	0.119	0.276	0.286	0.195
Conditional Entropy	0.464	0.504	0.466	0.504	0.484
Fusion Conditional Entropy	0.464	0.504	0.466	0.504	0.484
Fusion Multivariate Sample Entropy	0.301	0.323	0.361	0.360	0.336
Refined Maximum Predictability	**0.066**	**0.061**	**0.058**	**0.062**	**0.062**

**Fig 4:**
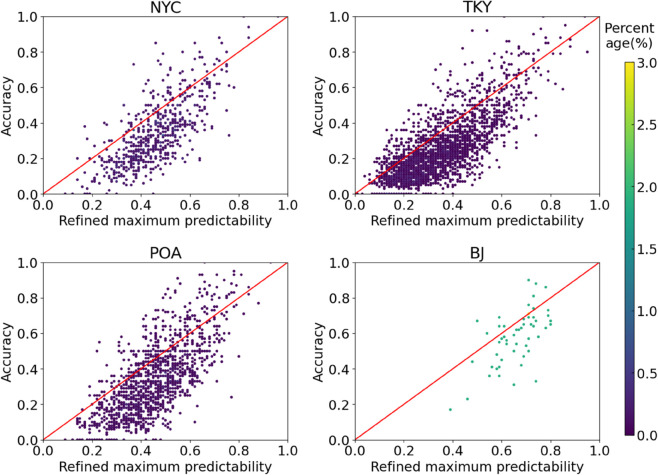
Correlation between the maximum accuracy and refined maximum predictability on real-world datasets.

*2) Case study.* To further validate the performance of the refined maximum predictability on real-world datasets, we compared the correlation between individual maximum prediction accuracy and the predictability results of different methods based on the four real-world datasets. The higher correlation indicates that the predictability calculation method can better estimate the individual regularity. We selected those users with more than 10 test samples since their prediction accuracies are more reliable. As shown in Table 5, the overall correlation between the maximum accuracy and refined maximum predictability was the highest, with correlation improvements ranging from 4.9% to 16.42% compared to the baselines. Specifically, the correlation values of refined maximum predictability ranged from 0.628 to 0.728, whereas the values of refined real entropy ranged from 0.549 to 0.694. Moreover, Fig 4 showed that the prediction accuracy increased with the refined maximum predictability, and its values were larger than the prediction accuracies for most users. These findings indicate that the refined maximum predictability can reflect individual regularity well, regardless of whether the input data consists of sparsely sampled check-ins or densely sampled GPS trajectories.

**Table 5:** Correlation between the maximum prediction accuracy and predictability on real-world datasets.

Method	NYC	TKY	POA	BJ
Real Entropy	0.605	0.689	0.639	0.531
Refined Real Entropy	0.615	0.694	0.649	0.549
Conditional Entropy	-0.356	-0.552	-0.393	0.028
Fusion Conditional Entropy	-0.356	-0.552	-0.393	0.028
Fusion Multivariate Sample Entropy	0.409	0.539	0.502	0.521
Refined Maximum Predictability	**0.716**	**0.728**	**0.690**	**0.628**
Improvment	16.42%	4.90%	6.32%	14.39%

We also compared the efficiency of different maximum predictability calculation methods based on the NYC dataset, and the experiments were conducted on a computer equipped with 16 GB of memory and an Intel Core i7-8700 CPU. As shown in Table 6, the maximum predictabilities based on real entropy had the fastest computation speed, requiring only 0.67 ms to calculate the maximum predictability for one user. In contrast, the method based on fusion multivariate sample entropy was the slowest. Since the real entropy, refined real entropy, and conditional entropy only consider one or two types of knowledge, the proposed refined maximum predictability incorporates and calculates predictability for four different types of knowledge, cumulatively increasing the computational complexity. Thus, it took 5.40 ms to calculate the predictability for one user, enabling single-threaded computation of 185 users’ predictability per second, which is enough for practical application. In summary, the proposed method sacrifices minimal computational speed to deliver a far more valuable, comprehensive, and accurate predictability assessment that can effectively evaluate and improve location prediction methods in a way traditional methods cannot.

**Table 6:** The prediction speeds of different methods on NYC dataset.

Method	Time
Real Entropy	0.67 ms
Refined Real Entropy	0.67 ms
Conditional Entropy	1.85 ms
Fusion Conditional Entropy	2.42 ms
Fusion Multivariate Sample Entropy	110.16 ms
Refined Maximum Predictability	5.40 ms

### Applications of knowledge preference

Based on the predictability for different knowledge, we further analyzed individual spatiotemporal knowledge preference and evaluated prediction models on real-world datasets.

**Fig 5:**
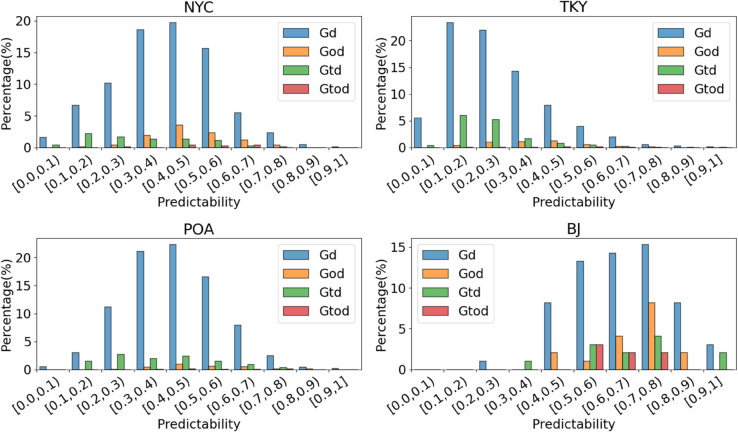
Distribution of the refined maximum predictability on real-world datasets.

*1) Dataset analysis.* The analysis of the maximum predictability is useful in many fields, such as traffic planning and management. The distribution of the proposed refined maximum predictability is shown in Fig 5. The datasets exhibited distinct predictability distributions. The BJ dataset was characterized by a high proportion of highly predictable individuals, with an average predictability of 0.671. Conversely, TKY had more low-predictability individuals, averaging 0.278. NYC and POA mainly consisted of moderately regular individuals, with averages of 0.413 and 0.434. Furthermore, the dominant predictability types were Gd and God for NYC and BJ, while Gd and Gtd were most prevalent in TKY and POA. These results reflect the underlying diversity of the datasets.

*2) Evaluations on prediction models.* We compared the refined maximum predictability with prediction accuracy to evaluate the knowledge utilization and selection for location prediction models, which can guide the design and improvement of prediction models. Finally, we applied the evaluation results to improve the Markov model.

**Fig 6:**
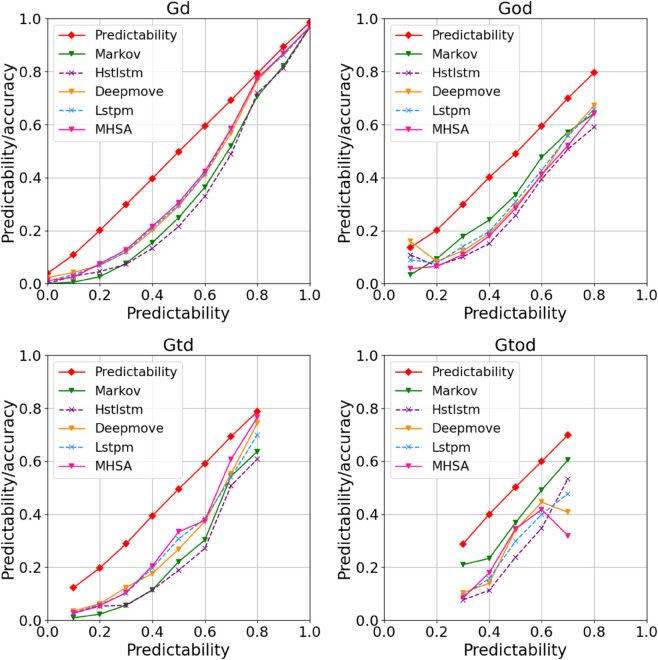
Distributions of the refined maximum predictability and prediction accuracy for different knowledge preference groups.

**Knowledge utilization.** The distributions of refined maximum predictability and prediction accuracy for different knowledge preference groups are shown in Fig 6. Overall, the prediction accuracy across all methods consistently falls below the refined maximum predictability, confirming that the proposed refined maximum predictability can represent the maximum potential predictability well. In the knowledge utilization, users in the God group with a predictability exceeding 0.8 exhibit larger gaps than other groups, highlighting significant room for improvement in utilizing origin-destination distribution knowledge. Regarding prediction methods, different approaches excelled in different areas: LSTPM and MHSA achieved the best prediction performance for the Gd and Gtd groups, while the Markov model performed best on the God and Gtod groups, which is consistent with its primary focus on origin-destination distribution knowledge. These findings demonstrate that our proposed method offers a more comprehensive evaluation of prediction methods, thereby guiding their future improvement and selection.

**Knowledge selection.** The importance of new knowledge can be evaluated based on the refined maximum predictability with new knowledge. To illustrate this, we used the next check-in time as an example. We extended the previous spatiotemporal knowledge to incorporate the distributions of the next check-in time-destination and next check-in time-origin-destination. The average maximum predictability then reached 0.426, 0.286, 0.469, and 0.694 in NYC, TKY, POA, and BJ, respectively. These are slightly higher than the predictability without considering the next check-in time. Therefore, the next check-in time is useful for location prediction.

**Improvement on prediction model.** The prediction models can be improved using the evaluation results. Here, we enhanced the Markov model using model evaluation results. Specifically, we selected knowledge corresponding to each preference group as prior information for location prediction, defaulting to origin-destination distribution knowledge when necessary. As shown in Table 7, the improved Markov model can improve the accuracy of Top 1 by 0.5 - 4.4 percentage points compared to the Markov model. Furthermore, the improved Markov model outperformed all baselines on the NYC dataset, while it slightly underperformed DeepMove, LSTPM, and MHSA on other datasets. This discrepancy may be attributed to variations in regularity distributions across these datasets. The Top 5 and Top 10 accuracy of the improved Markov model was close to that of LSTPM, MHSA, and DeepMove, indicating that the personalized selection and full utilization of spatiotemporal knowledge are important for location prediction. In addition, the improved Markov model does not require training, effectively saving training resources, and thus has substantial application potential.

**Table 7:** Performance of the location prediction models.

Model	NYC			TKY		
	Top1	Top5	Top10	Top1	Top5	Top10
Markov	0.232	0.485	0.576	0.178	0.355	0.417
Markov+	**0.251**	**0.561**	**0.673**	**0.192**	**0.424**	**0.519**
**Model**	**POA**			**BJ**		
	**Top1**	**Top5**	**Top10**	**Top1**	**Top5**	**Top10**
Markov	0.217	0.419	0.508	0.535	0.857	0.912
Markov+	**0.261**	**0.550**	**0.660**	**0.540**	**0.878**	**0.936**

In summary, the proposed refined maximum predictability estimates individual maximum predictability and spatiotemporal preferences well, supporting predictability analysis and location prediction model improvement, enabling: 1) Enhanced travel services, such as accurate trip time estimations and tailored recommendations for routes, parking, and Points of Interest. 2) Traffic management support. The maximum predictability dictates the controllable boundaries and potential effectiveness of management strategies. And the prediction results of high-predictability groups enable better assessment of future traffic conditions. Thus, on one hand, this information guides the macroscopic traffic management, such as traffic signal control, ramp metering, and the optimization of public transit routes and schedules. On the other hand, it facilitates personalized travel guidance: customized incentives can be used to induce highly predictable individuals to adjust their travel time or route, thereby alleviating congestion. Moreover, this methodology can be extended to other scenarios, such as sequence recommendation.

### Discussion

**Fig 7:**
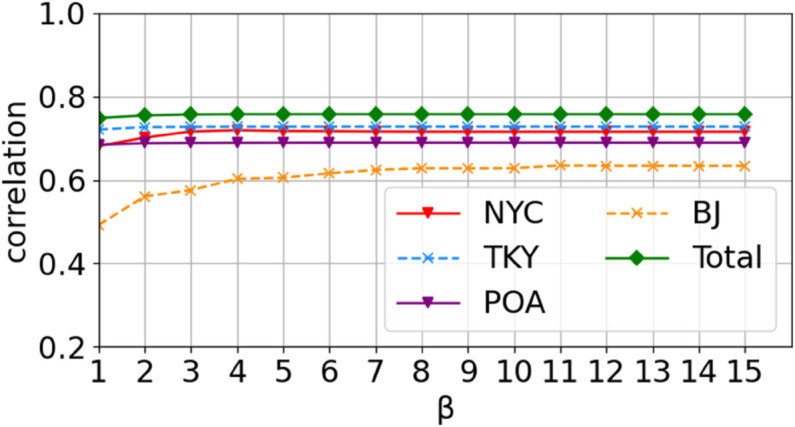
Correlations between the maximum accuracy and predictability for different β values.

*1) Threshold for non-repeating trips.* The non-repeating trip threshold, an essential component in calculating the refined maximum predictability, is determined by the average location frequency and a reduction factor (*β*). An excessively high reduction factor results in a low non-repeating trip threshold, which may overestimate the predictability of high-frequency but irregular trips. Conversely, a value that is too low results in a high threshold, which may underestimate the predictability of low-frequency but regular trips. To determine the optimal setting, we evaluated the impact of different *β* values on the correlation between maximum prediction accuracy and refined maximum predictability, using data from four diverse cities with varying sampling granularities. The experimental results are shown in Fig 7. Although the optimal range varied slightly by city, all datasets relatively performed best when the value was set to 8 or above, indicating a degree of universality for this range and leading us to adopt 8 as the optimal *β* value for these datasets. Furthermore, because the average location frequency for most individuals was less than 8 in these datasets, their non-repeating trip threshold stabilized at 1, making the overall predictability insensitive to further increases in *β* within this range. In summary, because the underlying distribution of individual travel patterns differs by city, the precise optimal *β* value range may vary slightly. Consequently, we recommend selecting a value of 8 or above for practical applications based on our empirical range, preferably opting for a larger value. Alternatively, the optimal value can be determined by reevaluating the correlation between location prediction accuracy and predictability in the specific new dataset.

*2) Spatiotemporal knowledge.* Here, we discussed the significance of considering the four types of spatiotemporal knowledge. As presented in Table 8, the correlation between maximum accuracy and predictability was the highest when considering all four types of knowledge. Specifically, when only considering destination distribution knowledge (d), the correlations ranged from 0.4923 to 0.7180. And the addition of temporal and origin-related knowledge to the refined maximum predictability provided a consistent improvement in correlation across all datasets. The BJ dataset showed the most significant overall increase in correlation when incorporating knowledge, rising from 0.4923 to 0.6281. Fig 5 also displayed that each knowledge preference group had a specific proportion of users in the four datasets. These findings indicate the crucial role of external knowledge in enhancing refined maximum predictability and that all four types of spatiotemporal knowledge should be considered.

**Table 8:** Correlations between the maximum accuracy and predictability when considering different types of knowledge on real-world datasets.

Method	NYC	TKY	POA	BJ
Refined Maximum Predictability -D	0.6664	0.7180	0.6842	0.4923
Refined Maximum Predictability -D, OD	0.7146	0.7276	0.6888	0.5994
Refined Maximum Predictability -D, OD, TD	0.7149	0.7277	0.6896	0.6247
Refined Maximum Predictability -All	**0.7160**	**0.7279**	**0.6896**	**0.6281**

## Conclusion

In this work, we focused on the estimation and applications of maximum predictability. And three problems persist, namely, incomplete consideration of spatiotemporal information, inadequate entropic measures, and the lack of utilizing predictability for detailed individual regularity analysis. To address these problems, we summarized spatiotemporal information and categorized it into four types. Then, we proposed a refined maximum predictability utilizing fusion knowledge and Shannon entropy. This method more accurately estimates predictability and identifies individual knowledge preferences, thereby improving group classification and prediction models. Our experiments demonstrated that the refined maximum predictability achieved a superior balance between spatial and temporal information, relatively outperforming classical methods by 68% in MAE on the simulation dataset. Moreover, the prediction model evaluations demonstrated the substantial potential of improving location prediction models with knowledge preference. Furthermore, the refined maximum predictability supports the inclusion or exclusion of various information types, allowing it to be generalized to mobility datasets with diverse spatiotemporal scales and information types, as well as extended to other prediction scenarios, such as sequence recommendation.
